# Inhibitory Effect of *Andrographis paniculata* Lactone on *Staphylococcus aureus* α-Hemolysin

**DOI:** 10.3389/fphar.2022.891943

**Published:** 2022-04-27

**Authors:** Xin Wang, Qiang Ma, Xiaohao Niu, Zhu Liu, Xinyun Kang, Yanni Mao, Na Li, Guiqin Wang

**Affiliations:** Veterinary Pharmacology Lab, School of Agriculture, Ningxia University, Yinchuan, China

**Keywords:** *Staphylococcus aureus*, toxicity factors, α-hemolysin, andrographolide, bioinformatics, molecular simulation

## Abstract

We investigated the effect of andrographolide (AP) on the hemolytic capacity of *Staphylococcus aureus* (*S. aureus*) isolated from our region. AP is a labdane diterpenoid isolated from the stem and leaves of *Andrographis paniculata*. The *hla* gene from 234 *S. aureus* strains and the quality control standard strain ATCC29213 in dairy cows in some areas of Ningxia was analyzed. Evolutionary analysis, homology modeling, and functional enrichment annotation of α-hemolysin Hla detected from our region were performed through bioinformatics. The hemolytic ability of *S. aureus* isolates from the region was examined using the hemolysis test, and the effect of AP on *S. aureus* was quantified. Moreover, the effect of AP on the transcript levels of *hla* and genes highly related to *hla* (i.e., *clfA* and *fnbA*) was examined through fluorescence quantitative PCR. The mode of action of AP on the detected Hla was analyzed through molecular docking and dynamic simulation. The results showed that *S. aureus* in our region has a high rate of *hla* carriage. The hemolytic activity of strains NM98 and XF10 was significant, and ATCC29213 also exhibited some hemolytic activity. AP could inhibit the expression of Hla and its related proteins by downregulating *hla*, *clfA*, and *fnbA* transcript levels, which in turn attenuated the *S. aureus* hemolytic activity. Meanwhile, the AP molecule can form three hydrogen bonds with residues ASN105, SER106, and THR155 of Hla protein; bind with PRO103 through alkyl intermolecular forces; and form carbon hydrogen bonds with LYS154, reflecting that the AP molecule has a comparatively ideal theoretical binding activity with Hla protein. Among them, PRO103 and LYS154 are highly conserved in Hla protein molecules and play pivotal roles in the biological functions of Hla, and their binding may affect these functions. Their binding may also prevent the conformational transition of Hla from a monomer to an oligomer, thus inhibiting Hla hemolytic activity. This study offers a molecular basis for use of AP as an antivirulence drug and new ideas for developing novel drugs against *S. aureus* infection.

## Introduction

*Staphylococcus aureus* is a highly virulent pathogen that is ubiquitous in the natural environment. It is causes a wide range of infections, from relatively minor skin lesions to life-threatening sepsis, posing a substantial threat to global health care (Kong et al., 2016; Tenover et al., 2019). Genomic plasticity confers *S. aureus* a strong ability to adapt to its environment, even in the presence of different drugs (Copin et al., 2018), consequently triggering the emergence of drug-resistant *S. aureus*. Recent increases in the number of multi-drug resistant strains with enhanced infectivity and virulence, such as methicillin-resistant *S. aureus* (MRSA) (Zhao et al., 2021), coupled with the low discovery rate of new antimicrobial drugs, has formed an urgent need for effective therapeutic strategies or new agents for *S. aureus* infections. Small molecules targeting bacterial virulence factors can inhibit the pathogenesis of *S. aureus*-mediated diseases, which is a promising therapeutic strategy (Wang et al., 2016; Ping et al., 2018; Xuewen et al., 2018; Tang et al., 2019). The strong pathogenicity of *S. aureus* is usually attributable to the relatively high expression of its virulence factors. One such virulence factor is α-hemolysin (Hla). Hla is secreted as a soluble protein monomer that binds to a membrane receptor and assembles into a heptameric complex, a pore-forming toxin capable of penetrating the plasma membrane. This penetration allows a large influx of Ca^2+^ and efflux of K^+^ ions and ATP (von Hoven et al., 2019), thereby causing cell membrane dissociation and destroying various host cells (including erythrocytes, alveolar epithelial cells, lymphocytes, monocytes, and macrophages). In addition, Hla causes apoptosis of monocytes, T-lymphocytes, and B-lymphocytes. Hla has been suggested to play a pivotal role in *S. aureus* pathogenesis (Berube and Wardenburg, 2013). The high virulence phenotype of the currently more popular USA300 strain is owing to the relatively high expression of several virulence factors, including *hla* (Montgomery et al., 2012). In addition, Hla-lacking mutant strains exhibit significantly diminished virulence against various animal models (Tkaczyk et al., 2013; Sampedro et al., 2014). Therefore, developing small molecule inhibitors for Hla-induced diseases is a promising therapeutic strategy.

Andrographolide (AP), a member of terpenoids (the most abundant and diverse group of natural products), is a multi-targeted molecule. Because of its complex structural features, such as low nitrogen and oxygen-rich, large number of sp3 hybridized carbon atoms, no aromatic ring structure, and polycyclic structure with aliphatic side chains, this molecule can effectively bind various biological targets and has varied pharmacological effects, including anti-inflammatory and antiviral (Tran et al., 2021). Moreover, AP is an α-alkylidene-β-hydroxy-γ-butyrolactone natural product that can covalently bind to nucleophilic residues (mainly cysteine residues) and irreversibly modulate target proteins, thereby enhancing their multi-targeting properties (Nguyen et al., 2015). However, the effect of AP on *S. aureus* α-hemolysin expression has not been investigated. We here found that AP significantly inhibited *S. aureus* hemolysis at low concentrations and highly significantly downregulated the transcript levels of *S. aureus hla* and its highly related genes *clfA* and *fnbA*. The inhibitory effect of AP on *S. aureus* α-hemolysin was subsequently explored through molecular docking, dynamic simulation, and binding free energy analysis.

## Materials and Methods

### Strains and Reagents

*S. aureus* strains used were the quality control strain ATCC29213 and the strains preserved in our laboratory (234 *S. aureus* strains, including strains NM98, XF10, XF12, Wld19, JY4, JY39, and JY41, were isolated from mastitis milk samples from dairy farms in different Ningxia areas in 2017–2018)). AP (Sigma-Aldrich, United States) was dissolved in DMSO (Solarbio Technology Co., Ltd., China) to prepare a stock solution at 40,960 μg/ml.

### Detection of *hla* Gene

The 234 *S. aureus* strains isolated and the strain ATCC29213 were removed from the −80°C refrigerator and screened using CHROMagar^™^ Staph aureus (CHRO Magar, France). Single purple-red colonies were selected for purification and culture. DNA was extracted using the TIANamp Bacteria DNA Kit (Tiangen Biotech Co., Ltd., China). Bacterial genomic DNA was used as the template, and *hla* primers (F: 5′-GGT​TTA​GCC​TGG​CCT​TC-3′; R: 5′-CAT​CAC​GAA​CTC​GTT​CG-3′), 2× Taq PCR Master Mix (Vazyme Biotech Co., Ltd., China), and deionized water (Tiangen Biotech Co., Ltd., China) to prepare a 20-μL reaction system. Gene fragment amplification was performed at 58°C, used as the optimum annealing temperature. The sequencing results were uploaded to the NCBI website for sequence comparison by using the BLAST tool.

### Phylogenetic Analysis of *hla* Sequences Detected in This Region

The *hla* sequences sequencing results were submitted to NCBI, and the gene sequences of known bacteria with high similarity to *hla* sequences were selected through BLAST homology search. The *hla* sequences detected in our region were used to construct phylogenetic trees by using CLASTALX 1.83 and MEGA X 10.2.6 and the neighbor-joining method. The gene trees were embellished through the ITOL online website (https://itol.embl.de/).

## Determination of Bacterial Hemolytic Capacity

### Qualitative Detection of Bacterial Hemolytic Capacity

*hla*-containing *S. aureus* cells were cultured to the log phase. Then, 5 μL of the bacterial solution was removed, added dropwise vertically on dried rabbit blood agar plates, and incubated overnight at 37°C to observe the size of the hemolytic rings. Meanwhile, the *hla*-containing *S. aureus* cells were cultured to the log phase, and the supernatant was collected after centrifugation (10,000 *g*, 10 min). Then, the phosphate buffered saline (PBS), bacterial culture supernatant, and rabbit defibrinated erythrocytes were added to a 1.5-ml centrifuge tube, gently inverted and mixed, placed in a biochemical incubator at 37°C, and incubated overnight. The morphology and number of erythrocytes were observed under a biological microscope (Motic China Group Co., Ltd., China).

### Quantification of the Effect of *Andrographis paniculata* Lactone on Bacterial Hemolytic Capacity

Several *S. aureus* strains with high hemolytic capacity were screened and cultured to the log phase. The bacterial solution, culture medium, and drug were mixed in 10-ml centrifuge tubes to achieve final drug concentrations of 0, 16, 32, 64, 128, and 256 μg/ml, respectively. After overnight incubation, the culture supernatant was collected through centrifugation (10,000 *g*, 10 min). Then, PBS, bacterial culture supernatant, and rabbit defibrinated erythrocytes were added to a 1.5-ml centrifuge tube, mixed gently and completely, and incubated for 15 min at 37°C. After incubation, the supernatant was centrifuged (5,500 *g*, 4°C, 1 min) and collected. The hemolytic activity was determined by measuring the absorbance of the supernatant solution at OD_543_ nm. The hemolytic activity of the control group supernatant without AP was regarded as 100% and was used as a reference to calculate the hemolysis ratio. Changes in the hemolytic capacity of *S. aureus* before and after AP treatment were analyzed through the hemolysis assay.

### Hla Protein Gene Ontology and Protein–Protein Interaction Enrichment Analyses

Gene ontology (GO) of Hla protein was performed using online databases such as DAVID (https://david.ncifcrf.gov/) and STRING (https://cn.string-db.org/), with a view to enriching and annotating Hla protein in terms of biological processes (BP), molecular functions (MF), and cellular components (CC). STRING was used to search for related proteins that interact with Hla protein and contains experimental data, text mining results from PubMed abstracts, and comprehensive data from other databases, as well as results predicted using bioinformatics methods. Protein–protein interaction (PPI) analysis was performed using this database. Cytoscape plugin Cytohubba was used to select the top 10 highly connected genes (Hub) genes by association.

### Effect of AP on *hla*, *clfA*, and *fnbA* Transcript Levels by Fluorescence Quantitative PCR

Two strains with more significant hemolysis and ATCC29213 were selected, and different AP concentrations were added to make the final drug concentration for each strain reach 0, 64, and 256 μg/ml. Their total RNA was isolated using TriZol reagent (Takara, Japan). The isolated total RNA was then treated with DEPC water (Tiangen Biotech Co., Ltd., China) and reverse transcribed using the RevertAid First Strand cDNA Synthesis Kit (Thermo Fisher Scientific, United States). PCRs were performed using the SYBR^®^Green Real-Time Fluorescence PCR Premix (Qiagen, Germany) in a 20-μL reaction system. The reactions were run using an ABI 7500 fluorescence quantitative PCR instrument (Applied Biosystems, United States), and the results were processed using the ΔΔCT method, in which *gyrB* was used as an internal reference gene. The primers used were *gyrB* forward, 5′-GCC​GAT​TGC​TCT​AGT​AAA​AGT​CC-3′, *gyrB* reverse, 5′-GAT​TCC​TGT​ACC​AAA​TGC​TGT​G-3′; *hla* forward, 5′-GGT​TTA​GCC​TGG​CCT​TCA​GC-3′, *hla* reverse, 5′-ACC​AGT​AAC​ATT​ACC​GTT​GAA​TCC​A-3′; *clfA* forward, 5′-CGA​TTC​TGA​CTC​CGA​CAG​TGA​TTC​C-3′, *clfA* reverse, 5′-CGC​TGT​CTG​AAT​CTG​AGT​CGC​TAT​C-3′; *fnbA* forward, 5′-AAG​ATC​AGC​AGA​TGT​AGC​GGA​AGC-3′, *fnbA* reverse, 5′-CTC​GTT​GTC​CTG​CAT​GAG​GTT​CTA​C-3′.

### Molecular Characterization of Hla Protein

The secondary structure was predicted and analyzed using the online software Predict Protein (http://www.predictprotein.org/) as well as Jpred4 (http://www.compbio.dundee.ac.uk/jpred4/). Homology modeling and conservativeness analysis were performed using AlphaFold (Q2G1X0, Identity: 99.7%), SWISS-MODEL (A0A0D3Q6S7), and the online server Consurf Web Serve (http://consurf.tau.ac.il/). The conserved amino acids and solvent accessibility were mapped to the three-dimensional structure of Hla protein using the software PyMOL to analyze the linkage between amino acid conservativeness and protein structure and function. Using the programs ProtScale (http://us.expasy.org/tools/protparam.htmL) and Compute PI/MW (http://www.expasy.org/cgi-bin/protscale.pl) in the online software ExPASy, the physicochemical property analysis was performed.

### Molecular Docking of AP and Hla Protein

The *hla* sequences were converted into amino acid sequences, and the tertiary structures of Hla protein were obtained through homology modeling by using AlphaFold (Q2G1X0, Identity: 99.7%) and SWISS-MODEL (A0A0D3Q6S7) (Tanaka et al., 2011), with the help of the molecular dynamics program GROMACS 2020.03 based on the AMBER99SB force field for 50-ns molecular dynamics simulations. The processed dominant conformation was used as the initial conformation for Hla protein docking. Small molecule structure files of AP were obtained from the PubChem database (https://pubchem.ncbi.nlm.nih.gov/). Energy minimization based on the MMFF94 force field was performed, and structure files were generated and used for docking. Subsequently, the AutoDockTools 1.5.6 program was used to hydrogenate the receptor protein Hla. In the meantime, the number of rotatable bonds of ligand molecules and docking boxes (Grid Box) were set. Semi-flexible molecular docking to receptor proteins was performed sequentially using AutoDock Vina and Smina programs. The obtained results were verified on the basis of the consistency of the aforementioned three procedures. The final results, which were selected with a balance of scoring and conformational fit, were visualized using PyMOL (https://pymol.org/2/) and Discovery Studio 2020.

## Molecular Dynamics Simulation and Binding Free Energy Analysis

### Molecular Dynamics Simulation

To further determine the stable binding of AP to Hla protein. After molecular docking, the Hla–AP complex was used as the initial conformation for 70-ns all-atom dynamics simulations performed using the classical molecular dynamics simulation software GROMACS 2020.03. The stability of the system was also measured on the basis of the root mean square deviation (RMSD), which indicates the degree of structural changes in protein molecules. Root mean square fluctuation (RMSF) was used to reflect the degree of freedom of motion of each atom in the molecule. The radius of gyration (Rg) reflects the degree of tightness of the protein before and after binding. The free energy landscape is used to characterize changes in free energy and the stability of conformation experienced by the protein during the simulation.

### Binding Free Energy Analysis

The g_mmpbsa calculation script (Kumari et al., 2014) for the Hla–Ap complex was applied using the Molecular Mechanics Poisson-Boltzmann surface area (MM-PBSA) method to calculate the binding free energy. This approach defines the binding free energy as follows:

$$\Delta {\text{G}}_{\text{bind}}{=\text{G}}_{\text{complex}}-{\text{G}}_{\text{free}-\text{protein}}-{\text{G}}_{\text{free}-\text{ligand}}$$

By definition, in solution, we can write the free energy of a molecule as follows:

$${\text{G}=\text{E}}_{\text{gas}}-{\text{TS}}_{\text{gas}}{+\text{G}}_{\text{solvation}}$$

where the solvation free energy can be further decomposed into polar and non-polar fractions as follows:

$${\text{G}}_{\text{solvation}}{=\text{G}}_{\text{polar}}{+\text{G}}_{\text{nonpolar}}$$

In the MM-PBSA method, energy, and entropy contributions of the gas phase are calculated according to the MM method:

$${\text{Egas}=\text{E}}_{\text{MM}}{=\text{E}}_{\text{bond}}{+\text{E}}_{\text{angle}}{+\text{E}}_{\text{dihedral}}{+\text{E}}_{\text{vdw}}{+\text{E}}_{\text{coulomb}}$$

$${\text{S}}_{\text{gas}}{=\text{S}}_{\text{MM}}$$

where E_bond_, E_angle_, and E_dihedral_ correspond to bond, bond angle, and dihedral interactions, respectively, and E_vdw_ and E_coulomb_ represent van der Waals and Coulomb electrostatic interactions, respectively.

The solvation energy in the MM-PBSA method contains two components: polar and non-polar solvation energies. The polar solvation energy is derived from the electrostatic interaction between the solute and solvent molecules. It is calculated using an implicit solvent model, where the solvent is considered a continuous medium, and the corresponding Poisson Boltzmann equation is linearized and solved numerically.

$${\text{G}}_{\text{polar}}{=\text{G}}_{\text{PB}}$$

The non-polar solvation free energy can be calculated using the empirical surface area method and is therefore also referred to as the surface solvation energy. The calculation requires knowledge of the solvent-accessible surface area A of the molecule with two empirical parameters γ and b:

$${\text{G}}_{\text{nonpolar}}{=\text{G}}_{\text{surface}}=\gamma \text{A}+\text{b}$$

Combining these aforementioned terms gives the free energy equation for MM-PBSA:

$${\text{G}=\text{E}}_{\text{MM}}-{\text{TS}}_{\text{MM}}{+\text{G}}_{\text{PB}}{+\text{G}}_{\text{surface}}$$

### Statistical Analysis

All assays were analyzed in triplicate. Statistical significance was analyzed using one-way ANOVA with IBM SPSS Statistics 25.0 statistical software, and a *p* value of <0.05 was considered statistically significant.

## Results

### *hla* Genetic Test Results

**FIGURE 1:**
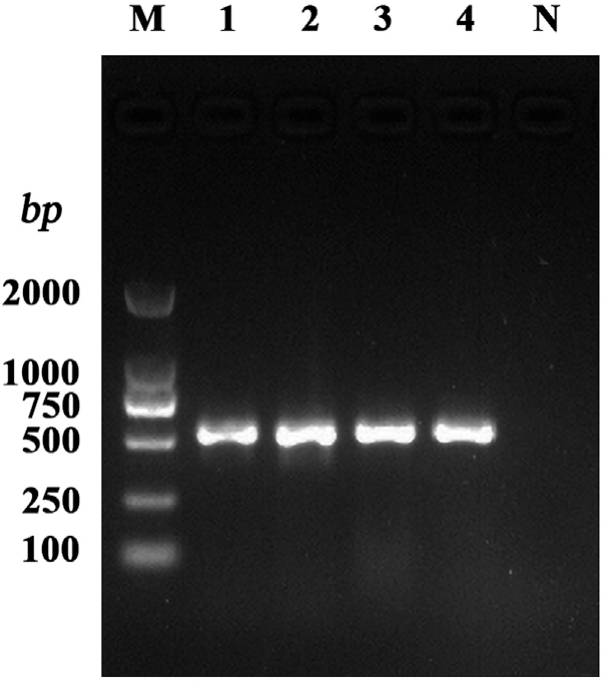
PCR amplification of the *S. aureus* virulence gene. Note: M: DL2000 DNA Marker; 1–4: test strains; N: negative control.

DNA of the 234 *S. aureus* strains and the quality control strain ATCC29213, isolated from mastitis milk samples from dairy farms in various Ningxia regions and kept in our laboratory, were used as template DNA for *hla* testing. The corresponding fragments of *hla* amplification are presented in Figure 1. The *hla* detection rate in the 234 strains was 98.29% (230/234).

### Phylogenetic Analysis of *hla* Sequences Detected in This Region

**FIGURE 2:**
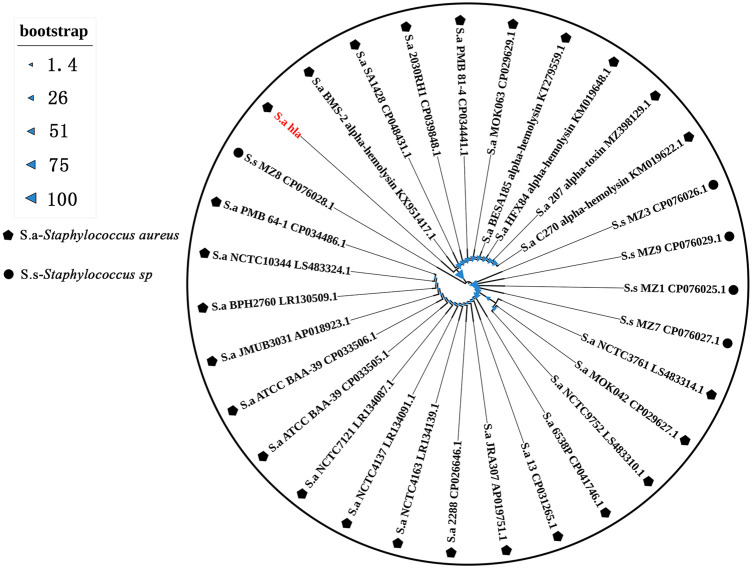
Phylogenetic tree based on the *hla* sequence.

The *hla* sequences detected in this region were uploaded to the NCBI database for blast comparison, and the results showed that the sequences had high homology with *S. aureus* SA1428, *S. aureus* 6538P, and *S. aureus* 13. The phylogenetic analysis showed that the detected sequences were the same as those carried by *S. aureus* BMS-2 α-hemolysin KX951417.1, *S. aureus* SA1428 CP048431.1, and *S. aureus* 2030RH1 CP039848.1, and the bootstrap value was 100 (Figure 2), indicating that the evolutionary tree constructed was highly reliable and reflects the high homology between the detected *hla* genes and those carried by the aforementioned strains.

### Qualitative Assay for the Hemolytic Capacity of *S. aureus*

**FIGURE 3:**
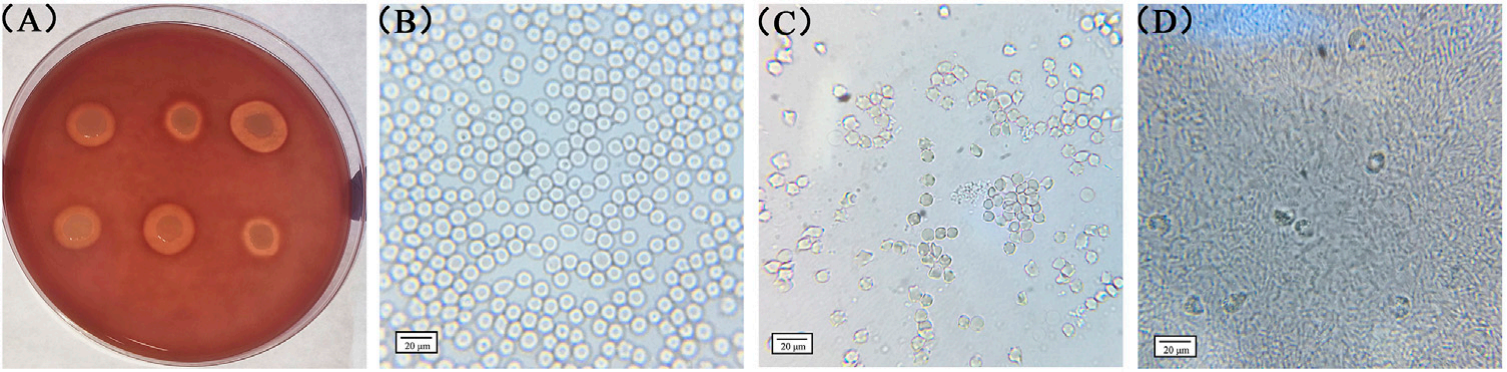
Rabbit red blood cells treated with different bacterial strains. Note: **(A)** presents the results observed on rabbit blood agar plates; **(B)**, **(C)**, and **(D)** present the results observed through biological microscopy.

To detect the hemolytic capacity of *S. aureus*, a hemolysis test was conducted using rabbit erythrocytes as they are more sensitive to the Hla hemolytic activity. Seven *S. aureus* strains with high hemolytic capacity were screened using rabbit blood agar plates, and their hemolytic capacity was further determined by microscopically observing the status of the rabbit blood erythrocytes treated with the bacterial solution. As shown in Figure 3A, a larger hemolysis ring indicates the stronger hemolysis ability of the strain. Further tests confirmed that strains with different sizes of hemolytic rings have different degrees of effects on the status of the rabbit blood erythrocytes. Figure 3B presents the blank control. In this case, rabbit blood erythrocytes with a diameter of approximately 7 μm were morphologically intact and more numerous in the field of view. Figure 3C shows rabbit blood erythrocytes treated with a strain with a medium-sized hemolysis loop; some erythrocytes in the field of view were morphologically incomplete and so appeared mutilated. Moreover, the number of erythrocytes in the field of view was considerably reduced compared with that in the blank control group. Figure 3D shows erythrocytes treated with a strain with a larger hemolysis loop; only few erythrocytes were scattered in the field of view, and the edges of the erythrocytes were rough and mutilated. Based on the aforementioned criteria, the strains NM98, XF10, XF12, Wld19, JY4, JY39, and JY41 were confirmed to be the most prominent hemolytic *S. aureus* strains in the region. The standard strain ATCC29213 was also found to have some hemolytic ability.

### Quantification of the Effect of AP on Bacterial Hemolytic Capacity

**FIGURE 4:**
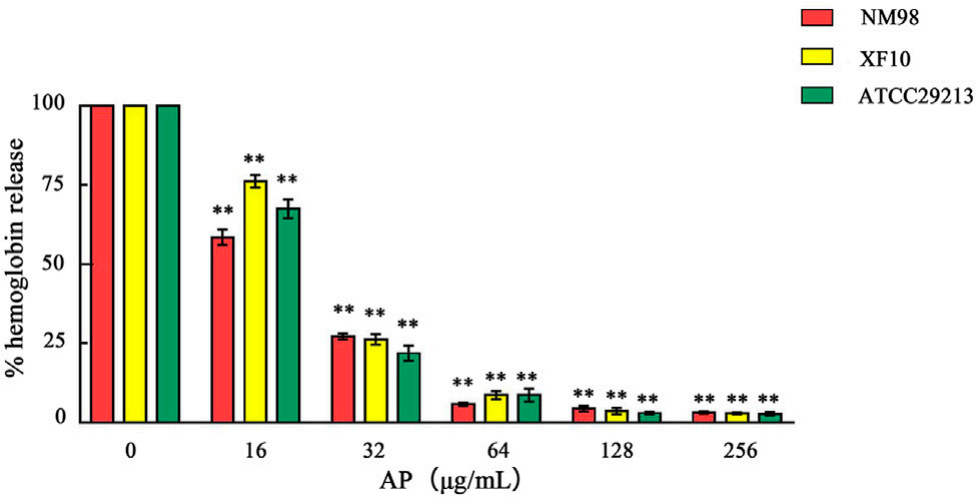
Effect of AP on *S. aureus* hemolysis. Note: NM98, XF10, and ATCC29213 denote different *S. aureus* strains; ** indicates *p* < 0.01 compared with the AP-free culture.

After qualitative detection of the *S. aureus* hemolytic ability, the two strains NM98 and XF10, which were more significant in hemolysis among the 7 strains, and the standard strain ATCC29213 were selected for quantitative detection. The effect of different AP concentrations on the hemolytic activity of the aforementioned three strains was further investigated through quantitative assays. The results showed (Figure 4) that the inhibitory effect of AP on the hemolytic activity of these three strains was concentration-dependent, with a highly significant (*p* < 0.01) decrease in hemolysis after AP treatment compared with the control without AP. Among them, the decrease in hemolysis was more in the NM98 strain than in the other two strains. Hemolysis was almost undetectable when the AP concentration reached above 64 μg/ml.

### GO and PPI Enrichment of Hla Protein

**FIGURE 5:**
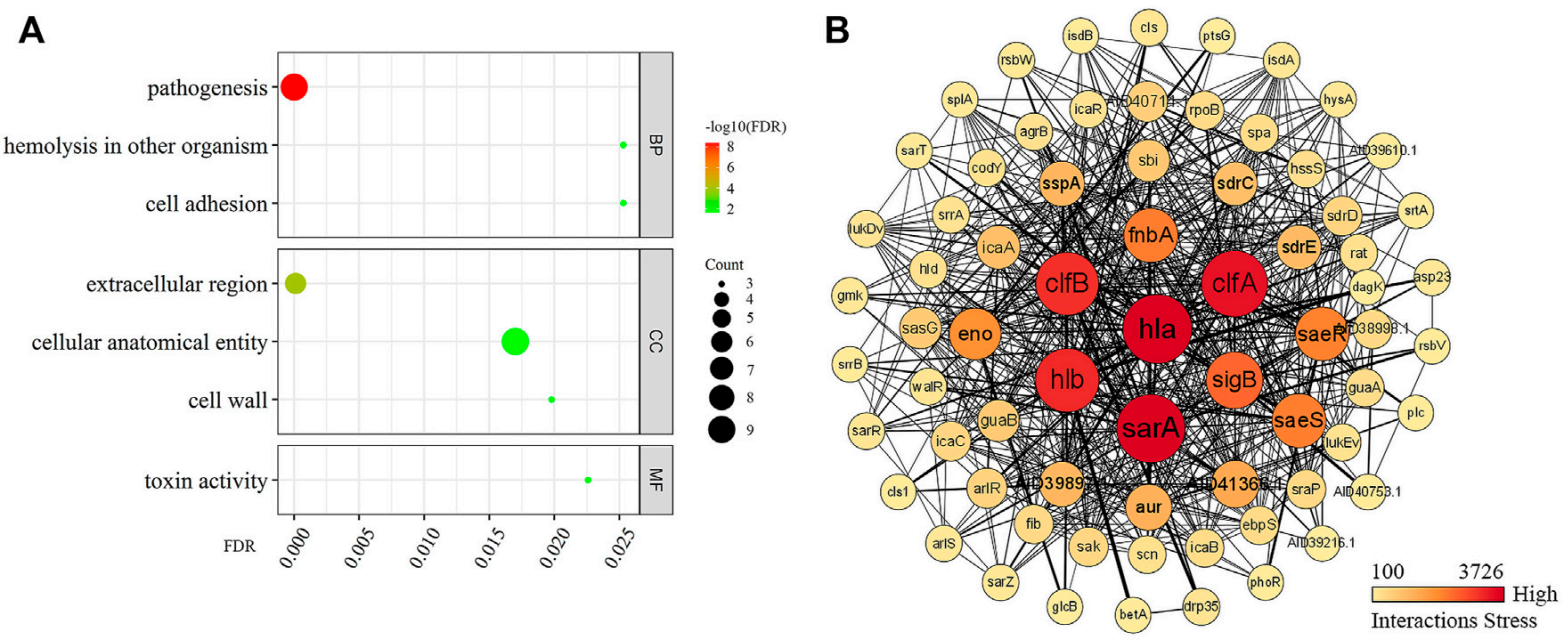
Enrichment analysis of GO and PPI of Hla protein. Note: **(A)** presents the GO enrichment analysis; **(B)** presents the protein–protein interaction; Darker colors indicate higher importance in the interaction network; the stronger the interaction between two proteins, the thicker the linkage.

GO revealed (Figure 5A) that Hla protein in the BP was highly associated with *S. aureus* pathogenesis, hemolysis, and colonization adhesion. In terms of CCs, Hla protein are mainly localized in the extracellular region and cellular anatomical entities. In terms of MF, Hla is mainly involved in toxin activity. The STRING database was used to synthetically mine the results from PubMed abstracts, synthesize data from other databases, and predict results using bioinformatics methods to synthetically mine other proteins that have interactions with Hla protein (Figure 5B) and filter the top 10 genes by using Cytoscape plugin Cytohubba Hub genes. The proteins encoded by *sarA*, *clfA*, *hlb*, *clfB*, *sigB*, *fnbA*, *saeS*, *saeR*, and *eno* are strongly associated with Hla protein. Hla protein is significantly associated and interact more closely with several global regulators, virulence proteins, and biofilm formation proteins in *S. aureus*.

### Effect of AP on *hla*, *clfA,* and *fnbA* Transcript Levels by Fluorescence Quantitative PCR

**FIGURE 6:**
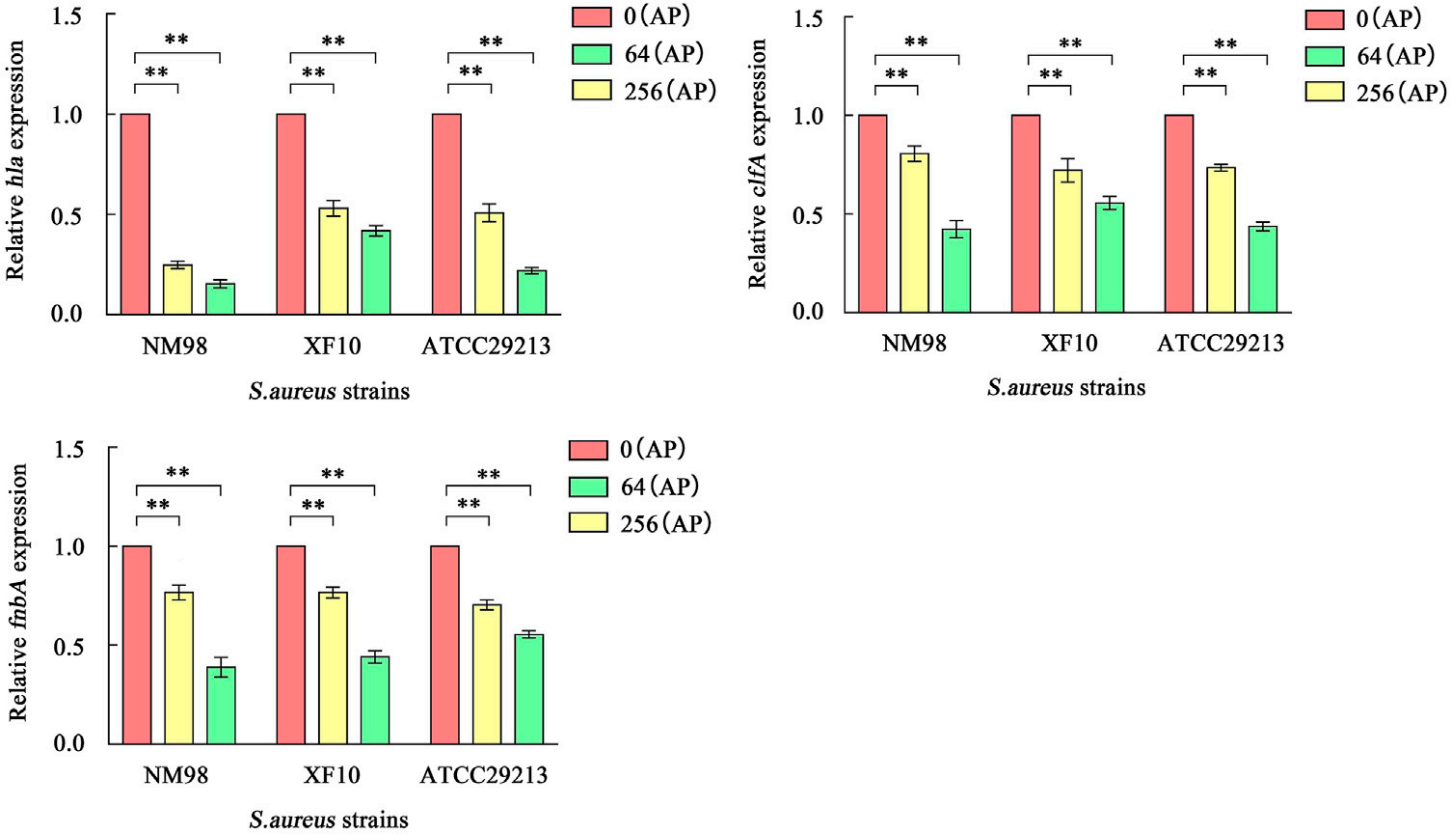
Effect of AP on hla, clfA, and fnbA transcription. Note: NM98, XF10, and ATCC29213 denote different *S. aureus* strains; 0 (AP), 64 (AP), and 256 (AP) denote 0, 64, and 256 μg/ml of AP, respectively; **p < 0.01 compared with the control.

Fluorescence quantitative PCR was used to detect the effect of AP at concentrations of 0, 64, and 256 μg/ml on the transcriptional levels of *hla*, *clfA*, and *fnbA* genes of *S. aureus*, aiming to investigate the transcriptional and translational activities of different AP concentrations on these genes*.* There are also some relevant differences. The results showed (Figure 6) that the transcript abundance of *hla* genes in NM98, XF10, and ATCC29213 was highly significantly downregulated after AP treatment at 64 μg/ml compared with the control (*p* < 0.01), with the NM98 strain showing the largest fold downregulation. Moreover, the transcript abundance of the three strains continued to be slightly downregulated when the AP dose was increased to 256 μg/ml. The aforementioned results are in good agreement with those of the hemolysis test analysis. The transcript abundance of *clfA* and *fnbA* genes in the aforementioned three strains were highly significantly downregulated (*p* < 0.01) after AP treatment at 64 and 256 μg/ml compared with the control. Among them, the NM98 strain exhibited a greater downregulation of the transcript abundance of *clfA* and *fnbA* genes after AP treatment at 256 μg/ml. In summary, AP can highly significantly downregulate the *hla*, *clfA*, and *fnbA* transcript levels.

### Molecular Characterization of Hla Protein

**FIGURE 7:**
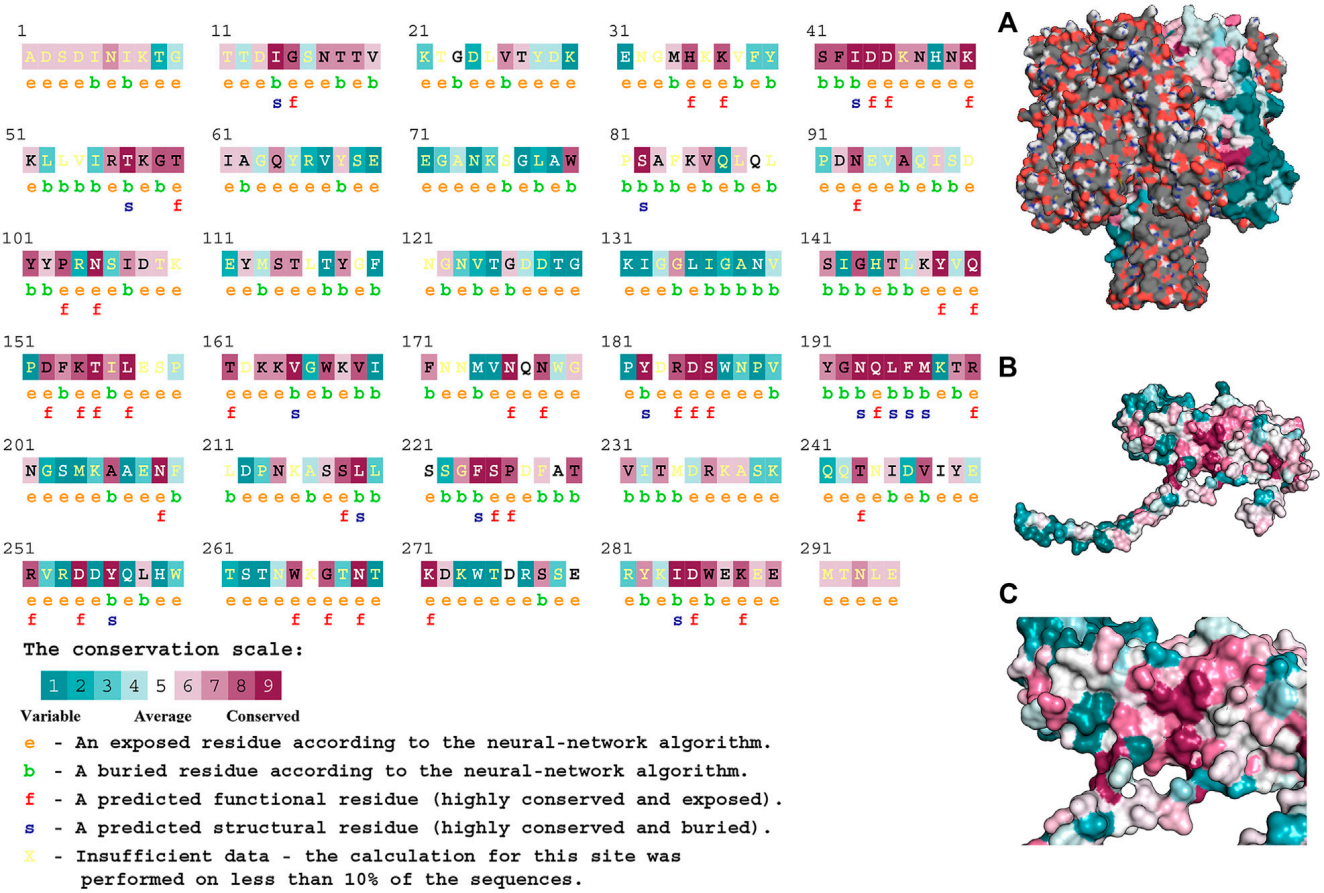
Structural characteristics of Hla protein. Note: **(A)** presents the heptameric structure of Hla; **(B)** and **(C)** presents Hla monomer.

As shown in Figure 7A, Hla protein can bind to form heptameric aqueous proteins, which are secreted by bacteria in the monomeric form (Figure 7B). The Hla protein contains 1.36% α-helix, 48.81% β-fold, and 49.83% irregular coiled. The association of conserved amino acids with structural stability and functional performance was analyzed through homology modeling and conservativeness analysis of Hla protein and projection of conserved assignments onto tertiary structures according to color (Figures 7B, C). The regions SER41-ASP45, ARG55-THR60, ASP152-LEU157, and TYR191-MET197 were found to be highly conserved, as revealed through the analysis. The prediction of amino acid solvent accessibility by the convolutional neural network algorithm reveals that multiple residues used to stabilize the highly conserved nature of the specific protein conformation are wrapped inside: ILE14, ILE43, THR57, SER82, VAL168, TYR182, ASN193, LEU195-MET197, LEU219 PHE224, TYR256, and ILE284. Amino acids located on the surface and highly conserved play crucial roles in biological functions, mainly ASP44, ASP45, LYS50, PRO103, ASN105, TYR148, GLN150, LYS154, THR155, LEU157, ASN176, ASP185, SER186, GLN194 ASN209, SER225, ASP254, ASN269, LYS271, and ASP285.

### Analysis of the Binding Mode of AP and Hla Protein

**FIGURE 8:**
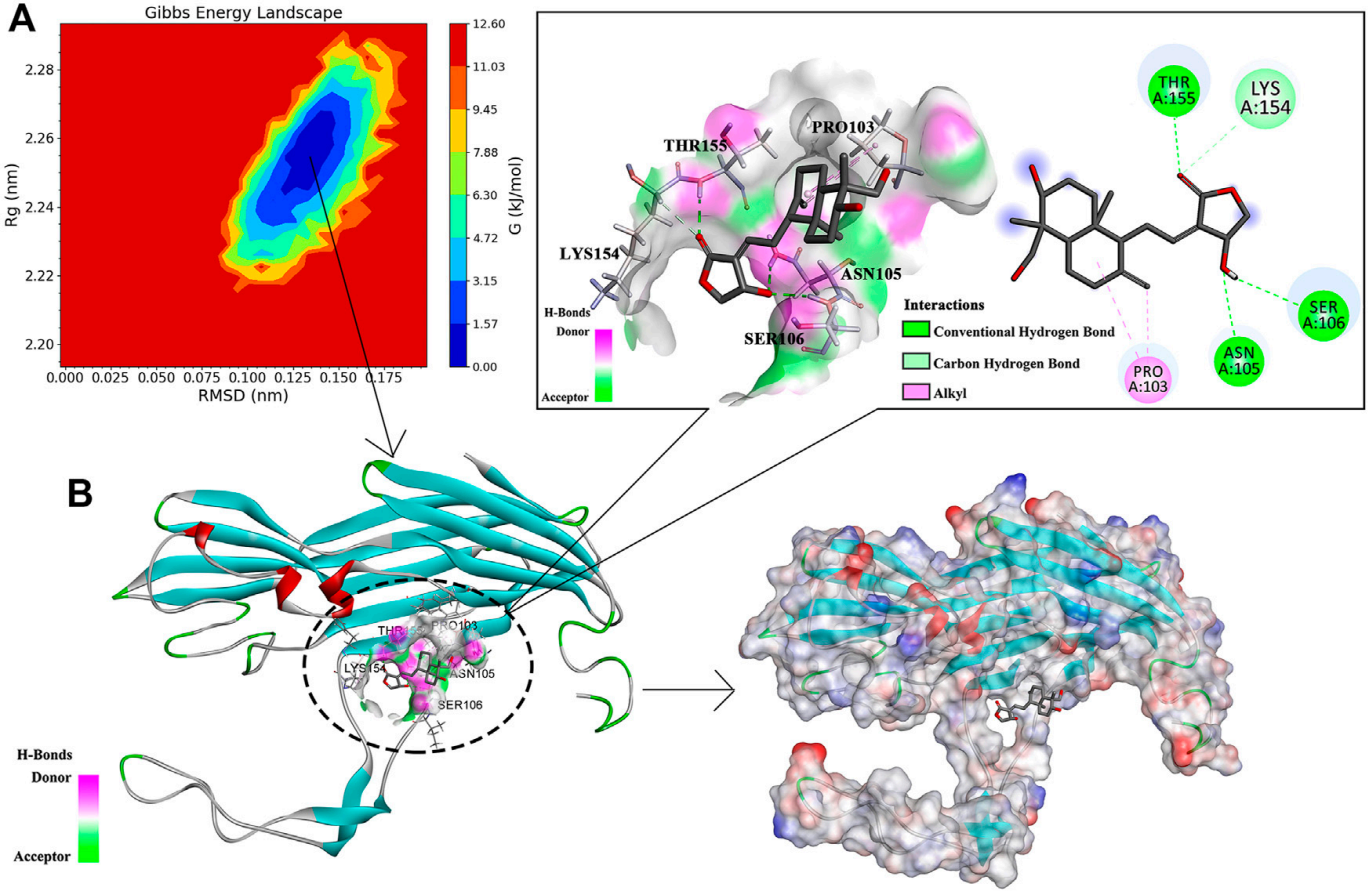
Free energy topography of the Hla–AP complex and its stable conformation. Note: **(A)** is the free energy topography of the Hla–AP complex; **(B)** is the stable conformation of the Hla–AP complex.

The data of three dimensions of RMSD, Rg, and Gibbs free energy were used to plot the free energy morphology (Figure 8A), obtain the time point of the low-energy structure, and finally determine the stable conformation of Hla–AP at 3650 ps (Figure 8B). AP could bind to the “triangular” region that connects the “stem” and “cap” regions in the Hla monomer structure. The triangular region of the Hla monomer plays a major role in the conversion of the Hla monomer to a heptamer (Walker and Bayley, 1995; Song et al., 1996). Therefore, we can infer that the binding of the triangular region to AP leads to a conformational change in the Hla monomer, probably hindering the formation of the Hla protein heptamer. Conformational visualization analysis revealed that the AP molecule can form three hydrogen bonds with residues ASN105, SER106, and THR155 of Hla protein; bind with PRO103 through alkyl intermolecular forces; and form carbon hydrogen bonds with LYS154. Among them, PRO103 and LYS154 are highly conserved residues that play pivotal roles in biological functions, and when AP binds to these proteins, the Hla biological functions may be affected. In this binding mode, AP binds to the Hla protein with lower energy, which fully reflects the reliability of AP binding to Hla. Therefore, a strong interaction between the aforementioned residues and AP is likely. These results are also confirmed through binding free energy analysis.

## Molecular Dynamics Simulation and Binding Free Energy Analysis

### Molecular Dynamics Simulation

**FIGURE 9:**
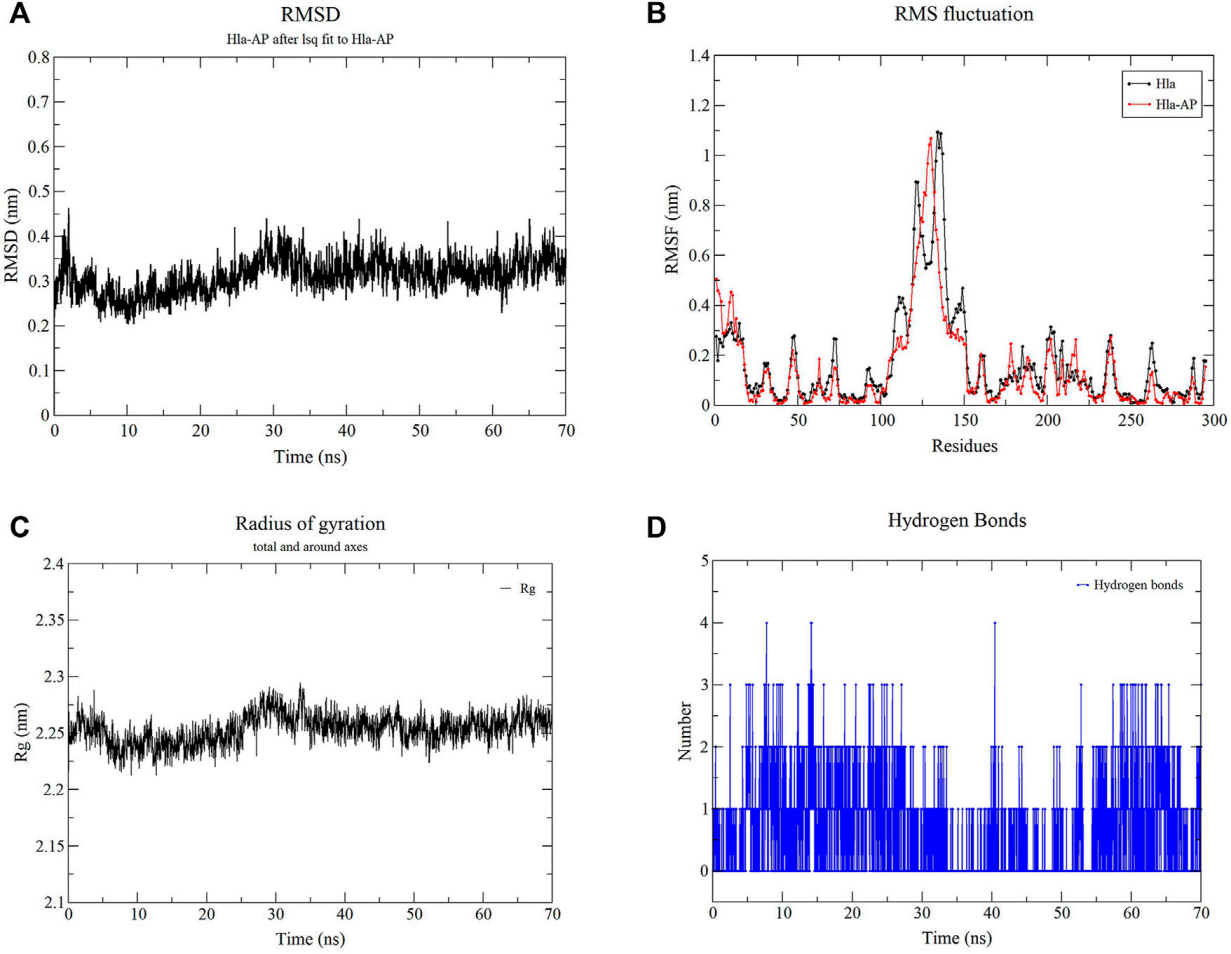
Molecular dynamics simulation of the complex of Hla–AP. Note: **(A)** presents the RMSD of the complex of Hla-AP; **(B)** presents the RMSF of the free Hla protein and the complex of Hla-AP; **(C)** presents the Rg of the complex of Hla-AP; **(D)** presents the number of hydrogen bonds formed between Hla and AP.

Molecular dynamics simulations of the Hla protein and its Hla–AP complex were performed for 70 ns by using GROMACS 2020.03. The stability of the complex conformation was measured on the basis of the RMSD of the complex, and as shown in Figure 9A, the complex reached equilibrium after 30 ns. The mean RMSD value was 0.328 nm during the molecular simulation of 30–70 ns, and the RMSD value fluctuated less in this time period, reflecting the stability of the system and the reliability of binding of AP and Hla. The RMSF was used to reflect the degrees of freedom of motion of the atoms in the molecule and to assess the flexibility of the residues (Figure 9B). The RMSF values of the Hla protein residues bound to AP were lower than those of the residues in the free Hla protein. The RMSF values of most residues were <3 Å, indicating that AP has a conformational stabilization effect on the Hla protein. Meanwhile, the Rg analysis also confirmed the aforementioned results; when Rg is smaller, the protein structure is more dense and more stable (Figure 9C). The Rg value of the Hla–AP complex was in the range of 2.20–2.29 nm during the simulation from the beginning to 30 ns, with a wave at 30 ns, and then gradually tended to be stable during the subsequent simulation (mean = 2.25 nm), further confirming the stability of AP and Hla protein. The role of hydrogen bonds in complex formation is also critical; the more the number of hydrogen bonds formed, the more stable the complex is. The number of hydrogen bonds formed between Hla and AP fluctuates between 1 and 2 during the 70-ns simulation (Figure 9D). Up to four hydrogen bonds can be formed, and a certain number of hydrogen bonds will further stabilize this binding conformation.

### Binding Free Energy Analysis

**FIGURE 10:**
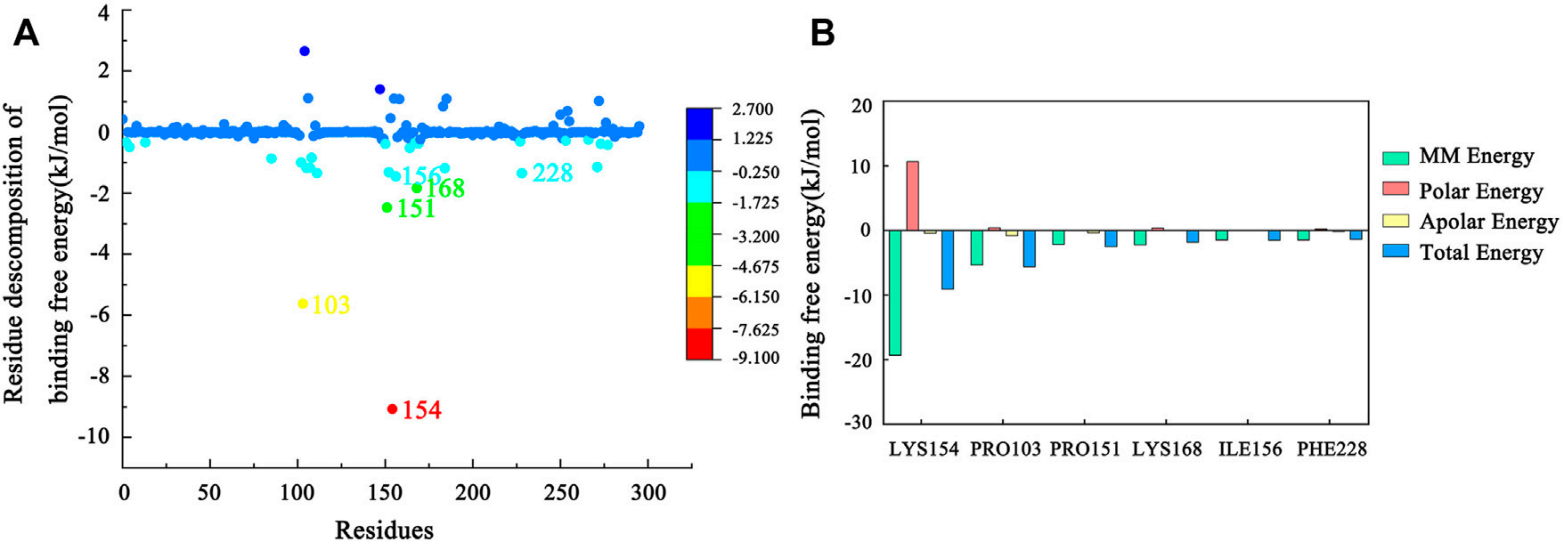
**(A)** MM-PBSA binding free energy decomposition for the interaction of each protein residue with the ligand. **(B)** Histogram showing the contribution of the six residues contributing most to the binding free energy of the complex of Hla–AP.

The total binding free energy of the Hla–AP complex during the simulation was calculated using the MM-PBSA method (Table 1), where the sum of van der Waals free energy, electrostatic free energy, polar solvation free energy, and solvent-accessible surface area free energy was the total binding free energy. The total binding free energy of the complex was −81.479 ± 13.502 kJ/mol (Table 1), where the binding of AP to Hla protein was influenced by the van der Waals free energy and polar solvation free energy. Meanwhile, the binding free energy was decomposed to each residue (Figures 10A, B), and the key residues in the region of AP to Hla protein binding were identified, in which the two residues contributing most to the binding were LYS154 and PRO103 (binding energy contributions: −9.07 ± 0.40 kJ/mol and −5.62 ± 0.20 kJ/mol, respectively), followed by PRO151 (−2.47 ± 0.18 kJ/mol), LYS168 (−1.83 ± 0.10 kJ/mol), ILE156 (−1.46 ± 0.07 kJ/mol), and PHE228 (−1.35 ± 0.11 kJ/mol).

**TABLE 1:** MM-PBSA-based total binding free energies along with its constituent energies for the selected bioactive molecules.

Complex	Van der waals energy (kJ/mol)	Electrostatic energy (kJ/mol)	Polar solvation energy (kJ/mol)	SASA energy (kJ/mol)	Total binding energy (kJ/mol)
Hla–AP	−96.687 ± 10.717	−39.997 ± 14.842	−67.412 ± 13.805	−12.207 ± 1.335	−81.479 ± 13.502

## Discussion

Antibiotics is one of the major advances of modern medicine. However, the widespread and long-term use of antibiotics has caused strong coercive pressure on bacteria, leading to a gradual increase in bacterial resistance (Powel et al., 2012). Clinically, multiple antibiotics or increased doses are required to treat infections caused by resistant bacteria, which only further induces stronger multi-resistant strains with many adverse effects on patients, who may eventually face a drug-free situation. Therefore, developing effective therapeutic strategies or new agents for *S. aureus* infections is urgently needed. *S. aureus* clinical infections are mainly due to the expression of multiple virulence genes. A therapeutic strategy that inhibits the expression of virulence factors and applies mild stress on bacteria (Rasko and Sperandio, 2010; Khodaverdian et al., 2013) is expected to be effective in addressing *S. aureus* clinical infections and drug resistance. Hla is the most crucial virulence protein of *S. aureus* and is a pore-forming toxin that disrupts host cell membranes, such as of erythrocytes and platelets, thereby causing cell lysis and eventually hemolysis (Song et al., 1996; von Hoven et al., 2019). Furthermore, the significance of Hla in *S. aureus* pathogenesis has been well documented in various animal disease models (Powers et al., 2012; Berube et al., 2014). Based on these results, we believe that Hla can be an ideal target protein for anti-*S. aureus* virulence.

We here found that *hla* in *S. aureus* from dairy cows in some areas of Ningxia is most closely related to that in strains such as *S. aureus* KX951417.1. Its detection rate was extremely high (98.29%), which is basically consistent with the *hla* detection rate reported by many researchers. A detection rate of 100% was also noted (Zhang et al., 2018; Achek et al., 2021), indicating that α-hemolysin is commonly found in *S. aureus*. This presence also proves the central role of α-hemolysin in *S. aureus* virulence from a certain perspective.

Numerous studies have shown that many naturally occurring active substances can attenuate the *S. aureus* hemolytic activity by inhibiting Hla expression, thereby achieving anti-*S. aureus* virulence (Ping et al., 2018; Xuewen et al., 2018; Tang et al., 2019). AP is a terpenoid derived from *Andrographis paniculata* that possesses various pharmacological effects such as anti-inflammatory, anti-viral, and anti-tumor (Tran et al., 2021). Banerjee et al. investigated the effect of AP on cell wall, protein, DNA, and RNA biosynthesis in *S. aureus* and found that AP could inhibit DNA, RNA, and protein biosynthesis but not cell wall biosynthesis (Banerjee et al., 2017). In the present study, AP significantly inhibited the hemolytic activity of strains NM98, XF10, and ATCC29213 at a low concentration (64 μg/ml). In addition, AP significantly downregulated the transcript levels of the *hla* gene as well as *clfA* and *fnbA*, which are highly related to *hla*, to suppress the expression of Hla and its related proteins, respectively, thus weakening the hemolytic activity of *S. aureus*. Clumping factor A and fibronectin-binding protein A encoded by *clfA* and *fnbA*, respectively, are adhesins belonging to the family of microbial surface components recognizing adhesive matrix molecules, which are key players in the adhesion colonization of *S. aureus* and biofilm formation (Foster et al., 2014; Speziale et al., 2019). Crismaru et al. showed that biofilms can protect bacteria to some extent from the action of drug molecules (Crismaru et al., 2011). From this, we infer that AP can also interfere with bacterial adhesion and thus inhibit biofilm formation, which facilitates the further efficacy of AP and allows it to reduce the hemolytic activity of *S. aureus*. In other experiments concerning this study, we determined the minimum inhibitory concentration (MIC) values and the effect of AP on the growth of the tested *S. aureus* strains in our region, namely strains NM98, XF10, and ATCC29213. No significant inhibitory activity of AP was noted against the studied strains (MIC >1024 μg/ml). AP at a concentration of 1024 μg/ml had almost no effect on the growth of the tested strains. Banerjee et al. found that the MIC value of AP against MRSA was 1000 μg/ml (Banerjee et al., 2017). This is similar to the MIC values of AP against strains from our region. Andrographiside sulfonamide (AS) salt has to been shown to significantly inhibit MRSA (MIC = 50 μg/ml) (Zhang et al., 2021). AS is a derivative of AP and its effectiveness against *S. aureus* is different from that of AP and the strains tested are also different. However, it is worth noting that in this study, AP did not attenuate the hemolytic activity of *S. aureus* by inhibiting its growth but by suppressing the transcriptional levels of *hla* and its associated virulence factors to inhibit the expression of related proteins. This also reflects an advantage that AP has over AS. AP can be effective in mitigating the emergence of drug-resistant strains by avoiding the stress of duress on *S. aureus* to a certain extent. In summary, AP can be used as a potential antivirulence strategy for mitigating α-hemolysin-involved *S. aureus* infections.

**FIGURE 11:**
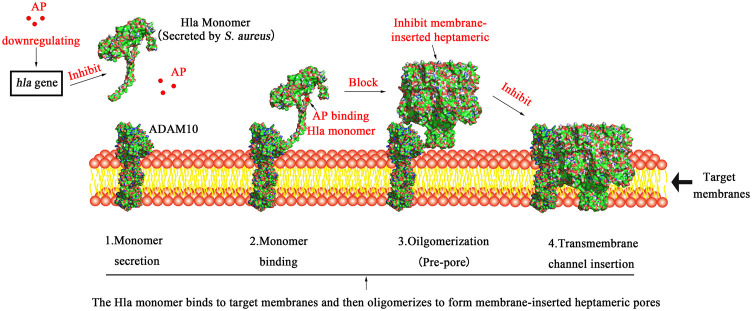
Schematic diagram of the mechanism underlying AP inhibition of Hla. Note: 1. Hla is secreted as a water-soluble monomer; 2. Hla binds to the transmembrane protein ADAM10, which is the cellular receptor for Hla; 3. Hla then oligomerizes into heptamers on the plasma membrane and forms a prepore; 4. Finally, transmembrane channels are formed; AP can inhibit the transcription of *S. aureus hla* gene at the genetic level to suppress the expression of Hla protein. On the other hand, AP binding blocks the conformational transition from monomer to oligomer in the critical triangular region of the Hla monomer, thereby inhibiting the cell lysis activity of α-hemolysin.

Drug design based on target proteins usually requires analysis of the structural features of protein–ligand complexes to determine the reliability of their binding. Molecular dynamics simulations are considered a crucial technique for analyzing protein–ligand interactions. We performed homology modeling of α-hemolysin Hla detected in our region using AlphaFold and SWISS-MODEL (Tanaka et al., 2011; Tunyasuvunakool et al., 2021). Moreover, phylogenetic analysis showed that *hla* detected in our region has high homology with known sequences in the database, which provides an important theoretical basis for Hla protein structural modeling in this study. This also lays the foundation for the subsequent study of the Hla functional structure. Meanwhile, the version of AlphaFold has been updated recently, and the new version predicts the protein structure more accurately (Jumper et al., 2021). It also provided the ideal initial protein structure for our experiments. In addition, we obtained the relevant free energy distributions using the MM-PBSA method, which is a combination of energy calculations based on molecular mechanics and free energy calculations from implicit solvent models. When correlated with molecular docking and dynamic simulation for comprehensive analysis, the protein–ligand binding mode can be studied exhaustively to determine the optimal binding site of the active compound to the target protein (Poli et al., 2020). The stem region of the Hla monomer binds to various naturally occurring active substances and prevents the self-assembly of heptamers across membrane pores, thereby inhibiting the cell lysis activity of α-hemolysin (Wang et al., 2015; Wang et al., 2016). In this study, we determined the stable conformation of the Hla–AP complex through comprehensive analysis of RMSD, RMSF, Rg, and hydrogen bond number, and Gibbs free energy data through dynamic simulation. Ultimately, we propose a different mechanism of inhibition than that reported above: AP binding blocks the conformational transition from monomer to oligomer in the critical triangular region of the Hla monomer, thereby inhibiting the cell lysis activity of α-hemolysin (Figure 11). In addition, the key residues in the binding of the Hla–AP complex, identified through residue decomposition analysis, were LYS154 and PRO103. Moreover, LYS154 and PRO103 are highly conserved residues in the Hla protein molecule that play crucial roles in biological functions and may affect the vital biological functions of Hla when AP is bound to it. This inhibition mechanism could lead to the development of new and more effective antimicrobial agents.

Studies have shown that the combination of natural active substances and conventional antibiotics can exhibit good synergistic effects against MRSA (Wang et al., 2021). Our experimental results showed that AP can inhibit the transcription of *S. aureus hla* and its related genes at the genetic level to suppress the expression of related proteins, thereby reducing the hemolytic activity of *S. aureus* on host cells. At the same time, AP can bind to the key “triangular” region of the Hla monomer secreted by *S. aureus* in a monomeric form, thus preventing the formation of the heptameric Hla protein (pore-forming toxin) and making it difficult for it to cause host cell lysis. Therefore, we speculate that the combination of AP with conventional antibiotics may exhibit better results against multidrug resistant strains such as MRSA, which will be the next direction of our research.

## Conclusion

AP showed a dose-dependent inhibition of the hemolytic activity of *S. aureus*. AP attenuated the hemolytic activity of *S. aureus* by downregulating the transcript levels of the *hla* gene and genes highly related to *hla* (i.e., *clfA* and *fnbA*), thereby inhibiting the expression of α-hemolysin and its related proteins. The inhibitory mechanism of AP against Hla was also proposed. AP could bind to the triangular region of the Hla monomer, resulting in conformational changes in the monomer, which might block the formation of the heptamer Hla protein. Moreover, AP also binds to the key residues LYS154 and PRO103, which are crucial for Hla biological functions, thus affecting the biological functions and weakening the cell lysis activity of Hla. Our study provides a new idea for investigating the hemolysis inhibition mechanism of other hemolysins and designing new and more effective antibacterial agents.

## Data Availability

The original contributions presented in the study are included in the article/Supplementary Material, further inquiries can be directed to the corresponding author.

## Abbreviations

AP, andrographolide; BP, biological process; CC, cellular component; FDR, false discovery rate; FEL, the free energy landscape; GO, gene ontology; MF, molecular function; MM-PBSA, the molecular mechanics poisson-boltzmann surface area; PDB, protein data bank; PPI, the protein-protein interaction; Rg, the radius of gyration; RMSD, the root mean square deviation; RMSF, the root mean square fluctuation; S. aureus, staphylococcus aureus.
